# The relationship of NM23 (NME) metastasis suppressor histidine phosphorylation to its nucleoside diphosphate kinase, histidine protein kinase and motility suppression activities

**DOI:** 10.18632/oncotarget.23796

**Published:** 2017-12-31

**Authors:** Imran Khan, Patricia S. Steeg

**Affiliations:** ^1^ Women's Malignancies Branch, Center for Cancer Research, National Cancer Institute, Bethesda, MD, USA

**Keywords:** NM23, metastasis suppressor, tumor metastasis, kinase, histidine

## Abstract

The NM23/NME gene was identified as a metastasis suppressor. It's re-expression inhibited cancer cell motility and suppressed metastasis, without effecting primary tumor size in multiple model systems. The mechanisms of NME suppression of motility and metastasis are incompletely known. Of particular interest, has been NME histidine 118 phosphorylation, involved in nucleoside diphosphate kinase (NDPK) and histidine protein kinase (HPK) activities. Using recently developed monoclonal antibodies to phosphohistidine, we have addressed the correlation of NME phosphohistidine with motility suppression, and distinguished the NDPK and HPK contributions. While general levels of NME correlated with its 1-phosphohistidine form in two cell line model systems, two exceptions were noted: Tumor cells actively migrating in scratch assays, even if expressing high levels of NME1, were low in its 1-phosphohistidine form. Site-directed mutagenesis of NME1 histidine 118 and proline 96 was examined by transfection experiments and partial purification of recombinant proteins. NME1^P96S^ overexpressing tumor cells exhibited high motility and migration phenotypes despite high 1-phosphohistidine content and NDPK activity; HPK activity using succinate thiokinase as a substrate was poor. The data suggest the importance of NME 1-phosphohistidine levels in potential mechanistic pathways of metastasis suppression and point toward the HPK activity of NME1 downstream of autophosphorylation.

## INTRODUCTION

The tumor metastatic process has defied therapeutic targeting, in part due to its intricate, dynamic regulatory pathways. Metastasis suppressor genes, genes that upon overexpression (or re-expression), significantly inhibit metastasis without effecting primary tumor size, constitute one avenue to dissect the metastatic process. Herein we focus on the NME/NM23 family of metastasis suppressor genes in one crucial component of metastasis, tumor cell motility *in vitro*. NME was credentialed by its reduced expression in highly metastatic melanoma cell lines as compared to equally tumorigenic, related, poorly metastatic melanoma cell lines [1]; overexpression in a variety of human and murine cancer cell lines reduced multi-organ metastasis without a significant effect on primary tumor size [2–12]. Simultaneously, a *Drosophila* form of NME, *abnormal wing discs* (AWD), was discovered, and controlled the differentiation of imaginal discs in larvae, linking development and metastasis [13]. A family of 10 NME genes have been identified in human [14].

The biochemical mechanism(s) of action of NME in tumor motility or metastasis suppression has been difficult to confirm, owing in part to its odd enzymatic activities, multiple binding partners and the presence of contaminants in some protein purifications. As far back as 1969, NME1 and −2 were reported to autophosphorylate on a histidine residue [15, 16]. This phosphorylation was undetectable under standard SDS-PAGE conditions as it is acid and heat labile; other obstacles to its characterization included the need for orthophosphate labeling, the lack of commercially available phosphohistidine standards in chromatography, and a lack of an antibody to phosphohistidine. NME phosphohistidine contributes to two enzymatic activities: As a nucleoside diphosphate kinase (NDPK), NME reversibly removes the terminal phosphate of a nucleotide triphosphate, autophosphorylating on its own H118, and then transfers the phosphate to a donor nucleotide diphosphate [17–20]. Several pathways have been hypothesized to use the NME NDPK activity to suppress tumor motility and metastasis [21, 22]. As a histidine protein kinase (HPK), autophosphorylated NME transfers its phosphate to a substrate protein [23, 24]. For NME2, known substrates include β subunit of heterotrimeric G proteins (Gβ) [25], potassium channel KCa3.1 [26] and TRPV5 (a member of TRP channel family) [27, 28] and are all phosphorylated on a histidine residue. For NME1, *in vitro* assays demonstrated phosphorylation of histidine residues in substrate proteins including ATP citrate lyase and succinate thiokinase [29]. In addition, a NME1-histidine to substrate serine phosphorylation was reported for the Kinase suppressor of ras (KSR) protein [30]; because of the difference in bond energies this transfer would be unidirectional. The NME1 HPK pathway has been correlated with tumor motility suppression [31]. Technical advances are required to make this research practicable. In addition to its enzymatic activities, NME proteins bind to a plethora of cellular proteins [32–36] that could contribute to metastasis suppression.

A remarkable advance in the field was recently reported, the development of monoclonal antibodies to N1-phosphohistidine and N3-phosphohistidine by the Hunter lab [37]. In total cell lysate, NME was the predominate protein labeled with anti-N1-phosphohistidine [37]. With this tool and new protocols for western blots and staining, it is now possible to visualize 1-phosphohistidine NME and the relationship of NME 1-phosphohistidine to total NME, its enzymatic activities and regulation of tumor cell motility.

## RESULTS

### NME suppression of *in vitro* motility in two model systems

**Figure 1:**
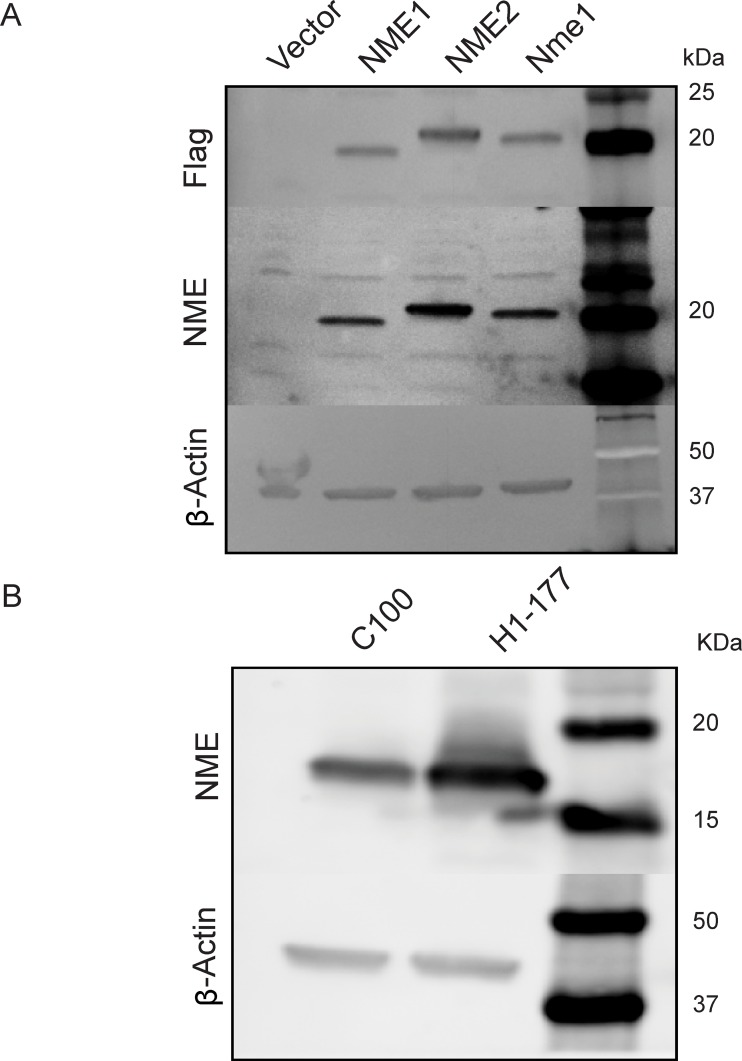
NME overexpression in two model systems **A.** Human MDA-MB-231T breast cancer cells were transfected with either vector construct, Flag-tagged human NME1, NME2 or mouse ortholog Nme1, and their overexpression was confirmed by western blot. β-actin was used as loading control. **B.** Human MDA-MB-435 cells previously transfected with vector control (C100) or NME1 (H1-177), were subjected to western blot analysis using β-actin as loading control.

Two sets of vector and NME transfected cells were used to characterize NME phosphohistidine expression. MDA-MB-231T triple-negative breast cancer cells were transfected with Flag-tagged human NME1, NME2, murine Nme1 or an empty vector (V), and pools of Flag-positive cells were collected (Figure 1A). The vector transfectant expressed an almost undetectable level of NME protein, while the Flag tagged overexpressed proteins ran at a slightly higher molecular weight than endogenous NME. For a second model system, freshly thawed, previously reported vector (C-100) and NME1 (H1-177) transfectants of the MDA-MB-435 line [3] were used. Protein expression trends were similar to those originally published (Figure 1B).

### 1-phosphohistidine levels in cell lysates

**Figure 2:**
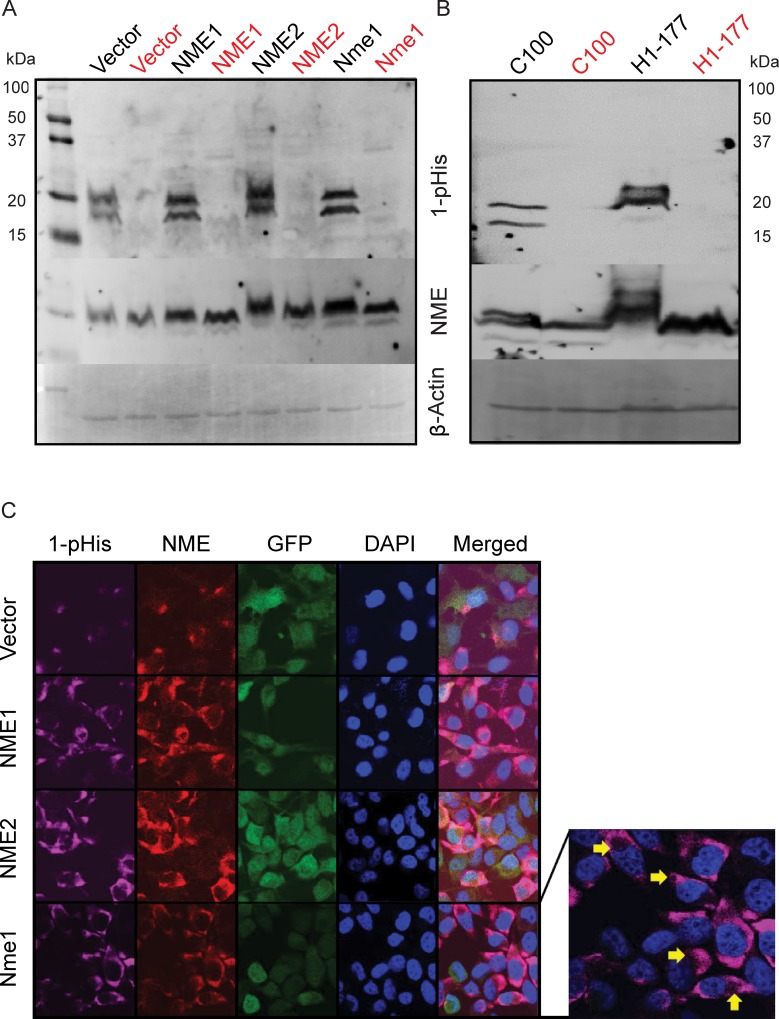
Correlation of total and 1-phosphohitidine NME level in cell lysates **A.** Cell lysates prepared from vector and NME overexpression (NME1, NME2 or mouse ortholog Nme1) in MDA-MB-231T cells were assessed for 1-phosphohistidine (1-pHis) level using anti-N1-phosphohistidine antibody on a basic gel with no heating. For specificity, replicate samples were heated at 95°C for 10 min and loaded next to each sample (red). **B.** MDA-MB-435 cells lysates from vector (C100) and NME overexpression (H1-177) were also assessed for 1-pHis in similar manner. Following stripping, both blots were re-probed with total NME and β-actin antibodies. **C.** For immunofluorescence, MDA-MB-231T cells with GFP co-expressing vector or NME overexpression were fixed in PFA and stained for 1-pHis using anti-N1-phosphohistidine antibody. Following 1-pHis primary incubation, cells were washed twice and were then stained with total NME1/2 antibody. Nuclei were visualized using DAPI (Blue). Images were captured at 65x magnification. Yellow arrows indicate vacuoles in 1-pHis staining highlighting acidic compartments.

NME was recognized as a 1-phosphohistidine containing protein based on initial discovery of an acid labile phosphorylation that was sequenced to its Histidine 118 [38, 39]. This histidine is mechanistically involved in two enzymatic activities, nucleoside diphosphate kinase (NDPK) and histidine protein kinase (HPK). The role of NME 1-phosphohistidine in NME biological suppression of tumor cell motility has remained largely unknown due to technical difficulties in working with phosphohistidine and an unexpected toxicity of H118 mutations in some overexpression experiments. A recently reported anti-N1-phosphohistidine antibody provided a technical advance in this field, and reported that the major 1-phosphohistidine expressing protein in cell lysates was NME [37]. Using anti-N1-phosphohistidine antibody, we asked if the 1-phosphohistidine content of cell lysates paralleled total NME protein levels. NME 1-phosphohistidine levels were quantified using a modified cell lysate and SDS-PAGE protocol at basic pH and without heating. As a control, samples were split and one half was heated, as 1-phosphohistidine is heat labile [37]. Figure 2A shows a 1-phosphohistidine blot of the MDA-MB-231T transfectants. NME proteins ran as a doublet of NME1 and −2. Overexpression of any NME protein in the MDA-MB-231T model system was accompanied by increased NME 1-phosphohistidine. Overexpression of NME1 is known to transphosphorylate NME2 and therefore 1-pHis intensity of both the doublet bands increased with NME1 overexpression. The blot was re-incubated with antibody to total NME and then β-actin as a loading control. Total NME protein, but not 1-phosphohistidine, was observed in heat treated samples, confirming the heat labile nature of the 1-phosphohistidine moiety. A similar experimental approach with MDA-MB-435 transfectants demonstrated similar trends (Figure 2B). As histidine can also become phosphorylated at N3 position, we assessed if NME became N3 phosphorylated using anti-N3-phosphohistidine antibody. No 3-phosphohistidine (3-pHis) band was detected in NME proteins (Supplementary Figure 1).

### NME and 1-phosphohistidine in cell cultures

Fuhs *et al*. also reported the use of the 1-phosphohistidine antibody for immunofluorescence staining [37]. As NME was by far the brightest 1-phosphohistidine band in total cellular lysates (Figure 2A-2B), it was reasonable to image total 1-phosphohistidine expression. Using a modified immunofluorescence protocol (all basic buffers, pH 9), total 1-phosphohistidine NME was compared to total NME (Figure 2C). Total NME in the vector transfectants was mostly cytoplasmic and concentrated in a few foci either near the cell surface or perinuclearly; 1-phosphohistidine was even more restricted than total NME, but overlapping its areas of greatest intensity. When any form of NME was overexpressed, cells exhibited heterogeneity in overall cellular levels and subcellular distribution of NME; 1-phosphohistidine levels also overlapped areas of greatest NME intensity. Small circular subcellular compartments were observed that could be acidic lysosomes (arrows). The specificity of 1-phosphohistidine immunostaining was demonstrated by incubating the slides in boiling citrate buffer for 10 min prior to staining, leading to loss of 1-phosphohistidine staining specifically (Supplementary Figure 2). Under standard culture conditions 1-phosphohistidine levels generally correlated with total NME expression.

### Correlation with NDPK activity and motility

**Figure 3:**
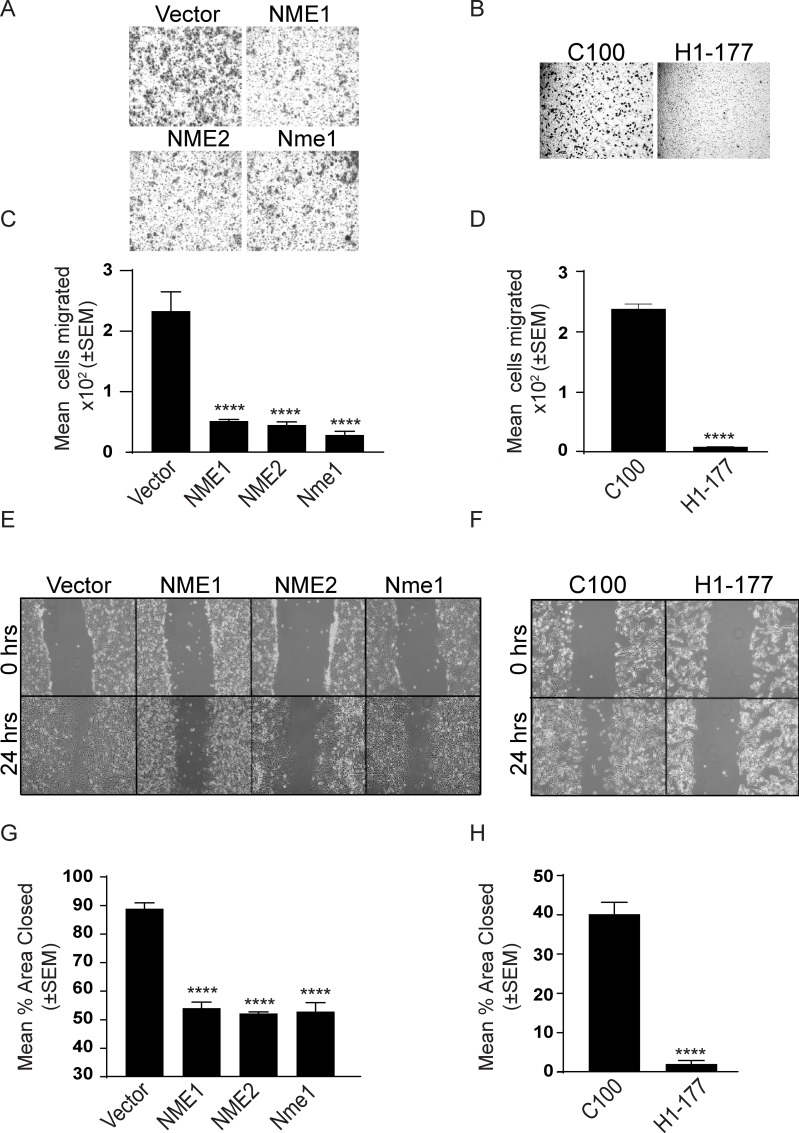
NME overexpression suppresses *in vitro* motility and migration NME overexpressing MDA-MB-231T (A, C, E, G) and MDA-MB-435 (B, D, F, H) cells were assessed for motility (A-D) and migration (E-H). **A**-**D.** Cells were permitted to migrate through a bovine collagen type I-coated 8μm membrane toward the chemoattractant (1% FBS) in a Boyden chamber. Cells migrated through membrane were photographed (A, B) and the mean number of cells migrated was quantitated using ImageJ (C, D). **E**-**H.** Cells were plated in 6-well plates and cultured for 24 hrs. A line was scratched with a 200-μm pipette tip and migration of cells into the scratch was photographed after 24 hrs (E, F). Percent area open was quantitated using ImageJ (G, H). All experiments shown are representative of 4 replicates performed (* *P* < 0.05, ** *P* < 0.01, *** *P* < 0.001, **** *P* < 0.001).

**Figure 4:**
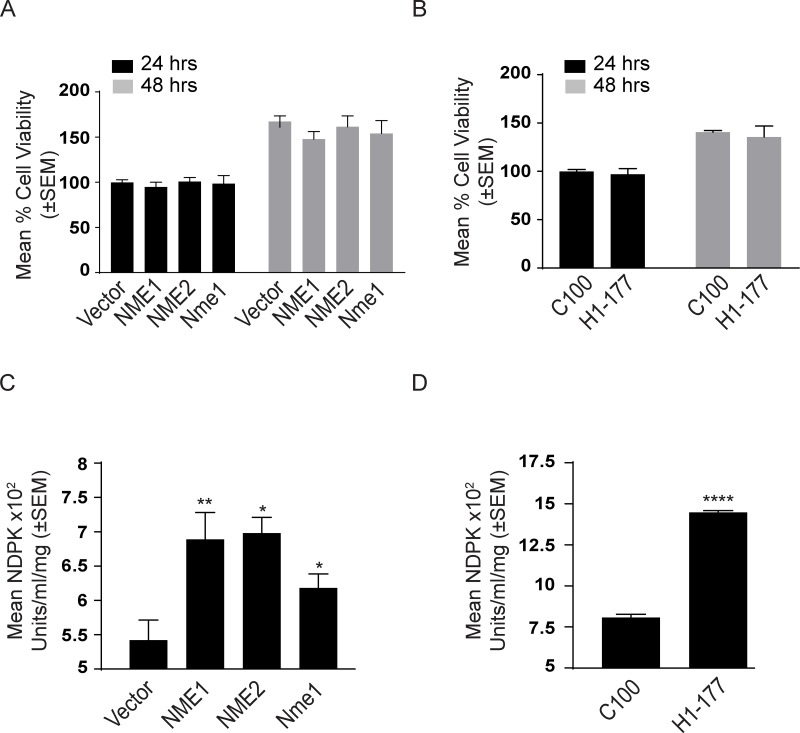
Characterization of NME transfectants Proliferation of NME overexpressing MDA-MB-231T **A.** and MDA-MB-435 cells **B.** after 24 and 48 hrs was assessed using AlamarBlue dye. The NDPK activity of NME overexpressing MDA-MB-231T **C.** and MDA-MB-435 **D.** cell lysates was quantified by a spectrophotometric assay for the formation of NAD (oxidized) from NADH (reduced) leading to the loss of OD at 340 nm. All experiments are representative of 4 replicates conducted.

One of the key *in vitro* correlates of NME function is suppression of tumor cell motility [40]. Using the combination of factors in fetal bovine serum (FBS) as an attractant, overexpression of NME significantly reduced motility in Boyden chamber assays (Figure 3A-3D) and migration in scratch assays (Figure 3E-3H). No effect was observed on proliferation (Figure 4A, 4B).

The NDPK activity of cell lysates was quantified by a spectrophotometric assay for the formation of NAD (oxidized) from NADH (reduced) leading to the loss of OD at 340 nm (Figure 4C, 4D, time courses on Supplementary Figure 3A-3C), When total cellular NDPK activity was quantified, 1-phosphohistidine and total NME levels were correlated with NDPK activity (Figure 4C, 4D). In cultures, 1-phosphohistidine expression paralleled total NME and NDPK activity, and was inversely related to tumor cell motility.

### Actively migrating tumor cells

**Figure 5:**
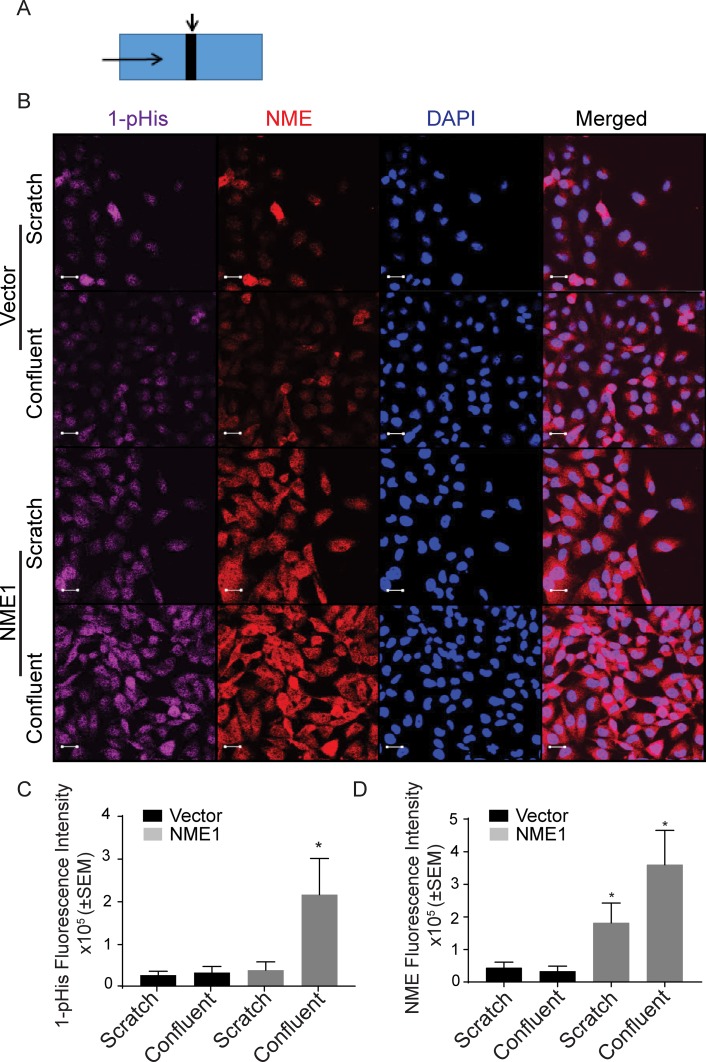
MDA-MB-231T migrating cells have low 1-phosphohistidine level regardless of total NME level Vector and NME overexpressing MDA-MB-231T cells were plated in chamber slides and cultured for 24 hrs. **A.** Schematic of culture. A line was scratched with a 200-μm pipette tip (Scratch is indicated by a downward arrow and movement of cells are indicated by horizontal arrow). Cells were permitted to migrate into the scratch for 24 hrs, while confluent cells distant from scratch remained nonmotile. **B.** After 24 hrs cells were fixed in PFA and immunofluorescence was performed for 1-phosphohistidine (1-pHis) and total NME1/2. Immunofluorescence staining was quantitated as total fluorescence intensity of 1-phosphohistidine (1-pHis) **C.** and total NME **D.** NME overexpressing cells in the scratch area did not exhibit strong 1-pHis positivity. Nuclei were visualized using DAPI (Blue). Images were captured at 65x magnification in the scratch and adjacent (confluent) area. Student's t test was performed between Vector-Scratch *vs* NME-Scratch and Vector-Confluent *vs* NME-Confluent.

**Figure 6:**
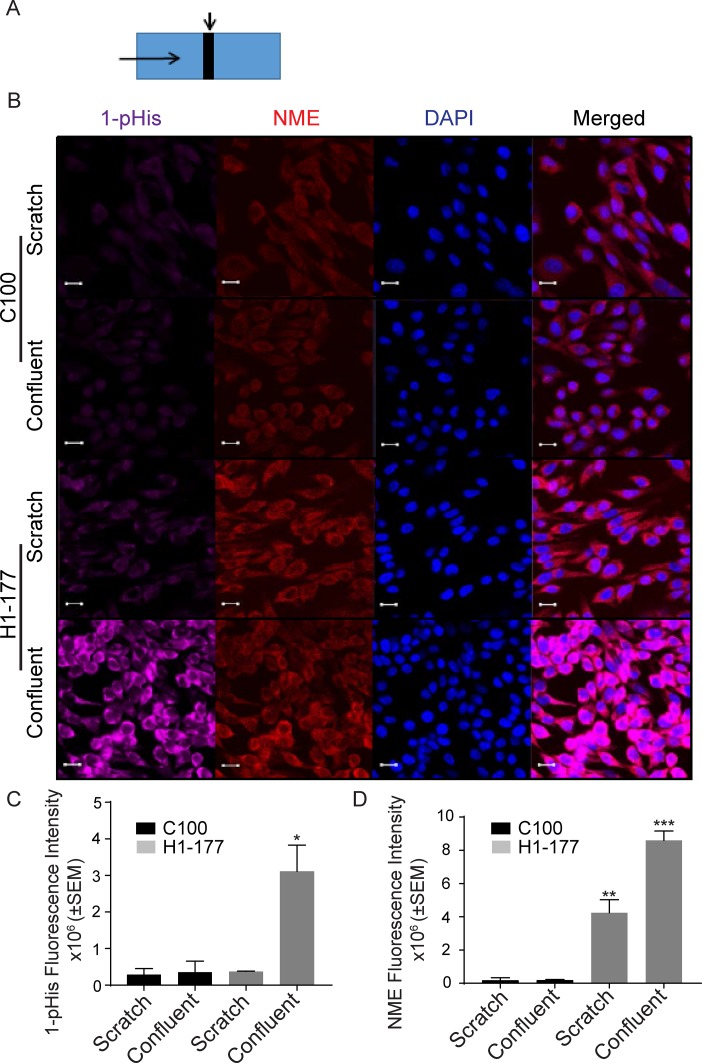
MDA-MB-435 migrating cells have a low 1-phosphohistidine level that does not correlate with total NME level **A.** Vector (C100) and NME1 (H1-177) overexpressing MDA-MB-435 cells were plated in chamber slides and treated as described in the legend for Figure 5A. **B.** After 24 hrs cells were fixed in PFA and immunofluorescence was performed for 1-pHis and NME1/2. Immunofluorescence staining was quantitated as total fluorescence intensity of 1-phosphohistidine (1-pHis) **C.** and total NME **D.** Both vector (C100) and NME overexpressing cells (H1-177) in the scratch area did not exhibit strong 1-pHis positivity. Nuclei were visualized using DAPI (Blue). Images were captured at 65x magnification in the scratch and confluent areas. Student's t-test was performed between Vector-Scratch *vs* NME-Scratch and Vector-Confluent *vs* NME-Confluent.

Cellular NME and 1-phosphohistidine levels were compared in migrating *versus* static conditions. Vector and NME1 transfected MDA-MB-231T and MDA-MB-435 cells were cultured in scratch assays as previously shown. Cells actively migrating in the scratch were compared to the mostly confluent monolayer distant from the scratch for total NME protein and 1-phosphohistidine (Figure 5A, 5B and Figure 6A, 6B). For the vector MDA-MB-231T transfectants, only occasional NME-positive cells were observed in either the scratch or confluent areas, and co-localized with 1-phosphohistidine. Upon enforced overexpression of NME1, total NME protein was higher in nonmotile and the few migrating cells. However, the 1-phosphohistidine level of the migrating NME overexpressing cells was comparable to that of the vector transfectants (Figure 5C, 5D). Nonmigrating NME overexpressing cells exhibited elevated 1-phosphohistidine levels consistent with data from cultures (Figure 2C). Similar trends were observed in MDA-MB-435 cells (Figure 6C, 6D). The data suggest that NME 1-phosphohistidine levels are regulated in tumor cell migration, and that total NME and its 1-phosphohistidine form are not correlated in active motility.

### NME mutations

To further explore the relationship of NME protein with its 1-phosphohistidine form, enzymatic activity and tumor cell motility, a series of previously reported NME mutations were re-derived in MDA-MB-231T cells. Histidine 118 is the site of NME autophosphorylation, and the H118F transfectants in the originally published MDA-MB-435 cell line failed to show NDPK or HPK activity above vector controls [41]. P96 is located on a loop structure of NME and is known as the *killer of prune* mutation due to its lethal interaction with the *Drosophila prune* gene [42–44]. Based on crystal structure and developmental studies in *Drosophila*, it is speculated that P96S mutation causes a change in NME conformation which possibly affects its binding partners or oligomerization leading to compromised/partial function. A NME1^P96S^ mutant transfectant in MDA-MB-435 cells was deficient in ice bucket HPK assays [31, 41, 45] but had NDPK activity consistent with wild type NME overexpression [41].

**Figure 7:**
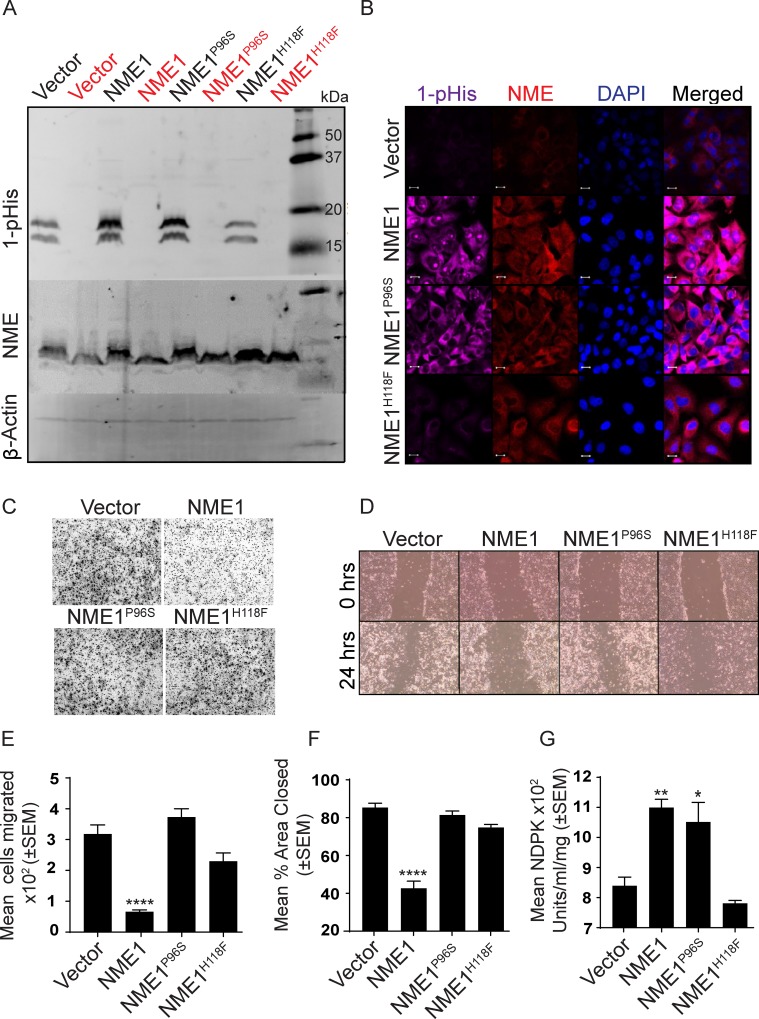
The NME^P96S^ site directed mutant distinguishes 1-pHis and NDPK activities from suppression of motility *in vitro* **A.** MDA-MB-231T cells stably expressing a vector, wild type NME1 or its P96S or H118F (NME1^P96S^ and NME1^H118F^) mutations were lysed and assessed for 1-pHis levels. Lysates were split into two, and one sample was heated (red). Blots were re-probed for NME1/2 and β-actin. **B.** 1-pHis and NME expression was also assessed by immunofluorescence in the above cells. Nuclei were visualized using DAPI (Blue). Images were captured at 65x magnification. **C.** Cells were tested for motility in Boyden chamber assay using 1% FBS as chemoattractant and motility quantitated **E**. **D.** Cells were tested for migration in scratch assay in 0.2% serum and migration quantitated **F**. **G**. The NDPK activity of lysates was quantitated using spectrophotometric assay. All experiments are representative of 4 replicates (* *P* < 0.05, ** *P* < 0.01, *** *P* < 0.001, **** *P* < 0.001).

MDA-MB-231T cells expressing a vector, wild type NME1 or its P96S or H118F (NME1^P96S^ and NME1^H118F^) mutations were prepared (Figure 7A, unheated samples). The 1-phosphohistidine levels of the transfectants is also shown on Figure 7A. NME overexpressing cells showed greater 1-phosphohistidine expression than vector controls as expected (Figure 7A and quantitation on Supplementary Figure 4A). The histidine mutant overexpressing cells exhibited 1-phosphohistidine levels approximating the vector transfectants, confirming the specificity of the antibody (Supplementary Figure 4A). The P96S transfectants exhibited 1-phosphohistidine levels comparable to wild type NME1 transfectants (Figure 7A and Supplementary Figure 4A). 1-phosphohistidine was heat sensitive (Figure 7A). Similar results were obtained for the level of 1-phosphohistidine and NME using immunofluorescence in NME wild type and mutants (Figure 7B and Supplementary Figure 4B, 4C). *In vitro* motility in Boyden chambers and migration in scratch assays are shown in Figure 7C-7F. Wild type NME overexpression significantly suppressed tumor cell motility (~80%) and migration (50%). For H118F overexpressing cells, a 28% diminution of motility to FBS was observed that was not statistically significant, and no decrease in migration was noted as compared to wild type NME1 in scratch assays (12%). The P96S mutant NME1 overexpressing cells were motility and migration competent at levels almost identical to vector control. The NDPK activity of the cell lysates paralleled NME1 1-phosphohistidine levels (Figure 7G). In summary, these data revealed a discrepancy between 1-phosphohistidine NME1 levels and biological function: P96S NME1 overexpressing cells exhibited high 1-phosphohistidine levels yet were motility competent.

**Figure 8:**
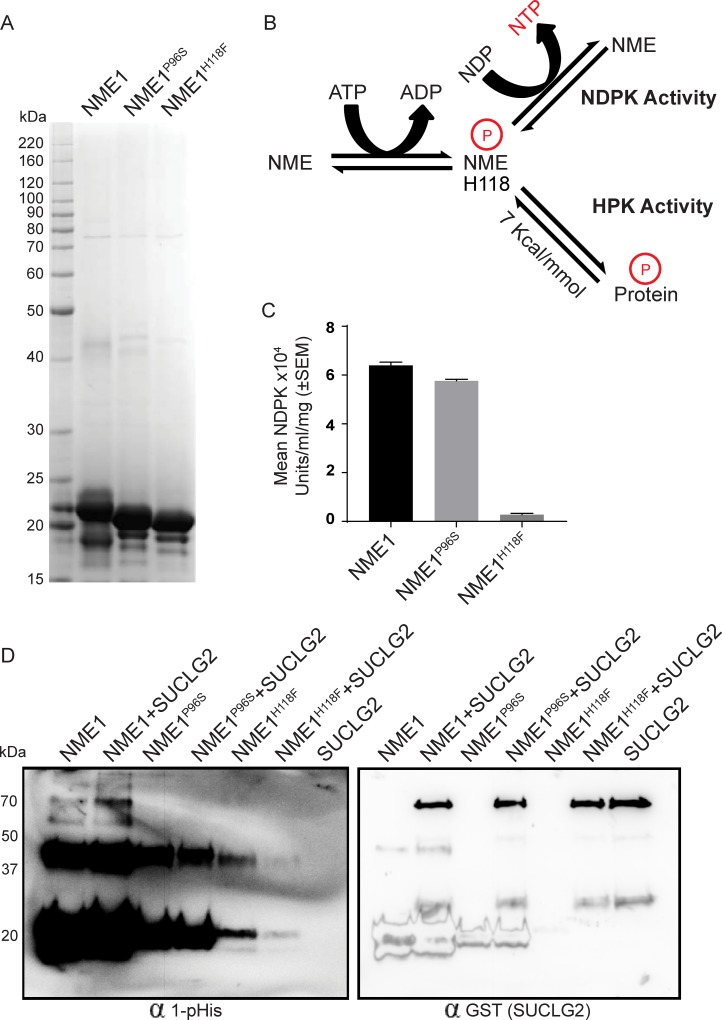
NME1^P96S^ recombinant protein provides a correlation of HPK activity with motility suppression **A.** Recombinant NME1, NME1^P96S^ and NME1^H118F^ constructs were His tagged, expressed in *E. coli* and partially purified by affinity purification using immobilized metal affinity chromatography. A coomassie stained 10-20% gradient SDS-PAGE gel of the partially purified proteins is shown. **B.** Diagrammatic representation of NME activities; NME1 is reversibly auto-histidine phosphorylated (H118) in presence of ATP. This phosphate, via a high energy phosphohistidine bond on NME1, is transferred reversibly to either a nucleotide diphosphate in NDPK activity or to a protein in HPK activity. **C.** The proteins were assessed for their NDPK activity by a spectrophotometric assay. **D.** The proteins were assessed for their HPK activity using SUCLG2 as their substrate. NME1, NME1^P96S^ or NME1^H118F^ (400 ng) were incubated with SUCLG2 (600 ng) in TMD buffer containing ATP for 30’ at room temperature. Reaction was stopped by adding 5x lysis buffer and 1-phosphohistidine western protocol was used for the detection of 1-pHis; after stripping, SUCLG2 (GST tagged) was detected by anti-GST antibody. Histidine phosphorylated SUCLG2 appears in NME1+SUCLG2 lane at ~70kDa. 1-pHis band at ~20 kDa and ~37 kDa are histidine phosphorylated monomer and dimer of NME1.

To further examine the relationship of NME1 mutations to enzymatic activities, recombinant NME wild type, P96S and H118F constructs were His tagged, expressed in *E. coli* and partially purified by affinity purification using immobilized metal affinity chromatography. Figure 8A shows a coomassie stained 10-20% gradient SDS-PAGE gel of the partially purified proteins. All proteins formed hexamers on native PAGE gel (Supplementary Figure 5) [46–48]. In Figure 8B the relationship of NME1 1-phosphohisitidine to its NDPK and HPK activities is outlined. A phosphate, via a high energy phosphohistidine bond on NME1, is transferred reversibly to either nucleotide diphosphate in NDPK or to a protein in HPK activity. The NDPK activity of the recombinant proteins was determined (Figure 8C) and trends matched that of the transfected MDA-MB-231T cell lysates (Figure 7G).

One of the clearest HPK substrates reported for NME1 *in vitro* is succinate thiokinase (Succinyl coenzyme A synthetase or Succinate-CoA ligase), as it is incapable of a histidine autophosphorylation [29]. Two succinate thiokinase forms are now recognized, alpha (SUCLG1) and beta (SUCLG2), which were obtained in partially purified form commercially. For the HPK assay, succinate thiokinase was incubated with recombinant NME1 and ATP as a triphosphate source; the resulting mixtures were resolved on a basic gel and probed for total and 1-phosphohistidine proteins (Figure 8D, left panel). For SUCLG2, a faint band at 70 kDa, the expected size of SUCLG2, was repeatedly observed in a reaction containing NME1 without added SUCLG2. Reprobing of the blot with anti-GST (SUCLG2) demonstrated that this was not recombinant succinate thiokinase (right panel). Importantly, the level of 1-phosphohistidine was increased at 70 kDa with addition of SUCLG2, indicative of NME1 HPK activity. Neither the NME1^P96S^ nor NME1^H118F^ recombinant proteins exhibited HPK activity to SUCLG2. Several controls further clarify the HPK assay: The recombinant SUCLG2-only lane in the right panel blot confirmed the mobility of this protein on the blot. A lack of any band in the left panel shows SUCLG2 has no autokinase activity. SUCLG1 was also tested as a possible substrate of NME1, however no increase in 1-phosphohistidine band (~70KD) could be seen with NME1 or in both mutants (Supplementary Figure 6A, B). As an additional control, 1-pHis blots of SUCLG1 and SUCLG2 were stripped and reprobed with NME antibody. NME1 predominantly formed monomers (~20 kDa) and dimers (~40 kDa), and did not form any oligomeric structures at 70 kDa which could overlap the 1-pHis band of SUCLG2 (Supplementary Figure 7). Similarly, a control gel with and without ATP for 1-pHis demonstrated that, without ATP, both NME1 and NME1^P96S^ did not autophosphorylate on 1-pHis (Supplementary Figure 8); NME1^H118F^ had no autophosphorylation with or without ATP (a very faint band could be endogenous wild type contamination). NME1 was also tested for HPK activity at the N3-histidine of both SUCLG1 and SUCLG2. No detectable phosphotransfer at 3-pHis occurred (Supplementary Figure 9). SUCLG2, being a substrate of NME HPK activity on 1-pHis, was also tested for phosphotransfer on other amino acids such as serine, threonine and tyrosine. No increase in phosphoserine, (Supplementary Figure 10A), phosphothreonine (Supplementary Figure 10B) or phosphotyrosine, (Supplementary Figure 11) was observed, with only autophosphorylation bands were visible.

Taken together, comparison of wild type and P96S NME1 proteins with *in vitro* motility suppression and enzymatic activities shows a clear disconnect. NME^P96S^ 1-phosphohistidine levels agreed with total NME1 levels and NDPK activity, but were deficient in HPK activity and suppression of tumor cell motility. The data suggest that the NME^P96S^ deficiency lies downstream of NME H118 autophosphorylation, possibly phosphorylation of key substrates needed for NME1 mediated motility suppression.

## DISCUSSION

A molecular understanding of the mechanistic pathways mediating tumor metastasis may lead to new therapies. The metastasis suppressor gene field may make original contributions to this field, as they function specifically in metastases and not in primary tumor growth (rev. in [49–51]). Of the many metastasis suppressor proteins and miRNAs, few have fully elucidated their mechanism(s) of action. The current work builds on a voluminous but inconclusive history of potential mechanistic pathways for metastasis suppression by the NME (NM23, nucleoside diphosphate kinase, AWD) family. Considerable evidence indicates that protein:protein interactions with NME modulate components of the metastatic process and *in vivo* metastasis ([52–54] as examples). The role of NME enzymatic activities has been less certain. Roles for the NME NDPK activity in the provision of certain nucleotides have been reported to contribute to dynamin function [55], oxidative stress [56], and endothelial contractility [57]. The NDPK activity of NME is not the only enzymatic activity emanating from its histidine 118 autophosphorylation, NME can also serve as a HPK. Histidine phosphorylated proteins may represent 6% of total protein phosphorylation in eukaryotes [58]. Recent data suggest that phosphohistidine status may influence the stability and activity of the EGFR receptor tyrosine kinase in the cancer microenvironment [59]. HPKs are poorly understood for technical reasons. The HPKs differ from well-studied kinases in that they form an autophosphorylated intermediate, histidine 118 in NME. This pathway has been best validated for NME2 in noncancer signaling including the beta subunit of G-proteins [60] and lymphocytic potassium channels [26]. Our work in the 1990s with Dr. Paul Wagner identified ATP citrate lyase [23], aldolase C [45], NME2 and succinic thiokinase [31] as *in vitro* HPK substrates of NME1 (although NME2 was likely bound), and limited mutational analysis suggested that this pathway may correlate with motility suppression. Progress in confirming these pathways and identifying metastasis-relevant substrates was impossible due to the acid and heat lability of the phosphohistidine bond, making even SDS-PAGE impossible. This field reopened in 2014 with the publication of monoclonal antibodies to 1- and 3-phosphohistidine, and methods for their use. NME1 and NME2 were the most prominent 1-phosphohistidine proteins in mammalian cell lysates [37]. We present herein the first analysis of NME 1-phosphohistidine expression in tumor cell motility.

In a comparison of vector and NME overexpressing transfectants of two cancer cell lines NME total protein often directly correlated with levels of NME 1-phosphohistidine observed on modified western blots or total 1-phosphohistidine in immunofluorescence staining. Controls established the heat and acid lability of 1-phosphohistidine and a lack of 3-phosphohistidine in agreement with the data of Fuhs *et al*. By immunofluorescence, 1-phosphohistidine staining overlapped areas of most intense NME staining. It should be noted that this staining could include multiple NME family members and other minor histidine phosphorylated proteins. This difference may suggest that only a portion of NME is histidine phosphorylated or may result from differences in the sensitivity of the two antibodies. There is no evidence for 1-phoshohistidine NME being limited to a particular cellular compartment. Two discordances were observed in 1-phosphohistidine NME and selected endpoints that were of greatest interest. The first was in immunofluorescence of actively migrating MDA-MB-231T cells in a scratch assay. Cells overexpressing total NME1 protein, that were migrating in the scratch area of a culture, were low in 1-phosphohistidine, comparable to vector transfectants, while nonmotile NME1 overexpressing cells away from the scratch in the same culture exhibited higher 1-phosphohistidine levels. This trend was observed in both MDA-MB-231T and MDA-MB-435 models. The trend was most stark in the latter model which may reflect the facts that overexpression of NME was higher, and that it was a clonal and not mixed population of transfectants. Thus, as cells overcome high NME1 expression and become motile, their histidine phosphorylation is decreased, suggesting less downstream NDPK or HPK activity.

A second discordance arose in the comparison of vector, wild type NME1 and NME1^P96S^ transfectants. Both cell lysates and partially purified recombinant proteins were examined. By western blots, the NME1^P96S^ transfectant exhibited high total and 1-phosphohistidine NME levels, comparable to the wild type protein. Similar data were observed in recombinant proteins. Both the lysates and recombinant proteins of the P96S transfectant exhibited high NDPK activity, consistent with wild type NME overexpression. Yet, the NME1^P96S^ transfectants did not suppress motility or migration *in vitro*, a discordancy of total and 1-phoshohistidine NME with motility suppression. A hypothesis to explain this apparent discrepancy would be that the HPK activity of NME, downstream of its autophosphorylation, was mechanistically involved in motility suppression. A HPK assay was set up using the anti-1-phosphohistidine antibody and partially purified recombinant NME proteins. Given our reliance on NME1, due to a literature on tumor motility suppression and site-directed mutagenesis, we chose a NME1 HPK substrate, succinic thiokinase, as it fails to histidine autophosphorylate *in vitro*. The SUCLG2 and not SUCLG1 form was histidine phosphorylated by wild type NME. SUCLG2 contains only one histidine at position 105, which would be the site of phosphorylation. Both the P96S and H118F mutant NME proteins were unable to kinase SUCLG2 *in vitro*. The data support the hypothesis that the HPK activity of NME1 is mechanistically linked to its motility suppressive capacity. The P96S mutation likely affects a part of the HPK activity downstream of autophosphorylation such as interaction with and phosphate transfer to the substrate. With these data, it will be of interest to identify the potential physiological substrates of NME1 as a HPK. Fuh *et al.* identified 630 histidine phosphorylated proteins by a modified mass spec methology, which could be substrates for NME1 [37]. In addition, it is possible that some of the many binding partners of NME1 may also be substrates. Another potential explanation for the data is that the 1-phosphohistidine form of NME1 has altered protein:protein binding activities, also affected by the P96S mutation. Further work is aimed at confirming a role for these activities in metastasis *in vivo*, and in other published biological processes such as development [61, 62].

## MATERIALS AND METHODS

### Cell culture conditions and treatments

Human triple negative breast cancer cell line MDA-MB-231T (A subline of human MDA-MB-231 cells, generously provided by Dr. Zach Howard [63], Laboratory of Immunoregulation, National Cancer Institute, Bethesda, MD) and MDA-MB-435 tumor cells were cultured in Dulbecco's Modified Eagle Medium (DMEM) (Invitrogen, Grand Island, NY) supplemented with 10% FBS in a humidified 37°C incubator at 5% CO_2_. MDA-MB-231T cells have been authenticated by our laboratory to the ATCC MDA-MB-231 line by short tandem repeat profiling.

### Transfection and generation of stable cell lines

MDA-MB-231T cells overexpressing NME1, NME2 and Nme1 was generated using lentivirus system (10^7^-10^9^ TU/ml, C-Flag-SV40-eGFP-IRES-Puromycin; GeneCopoea). 5×10^4^ cells/well were plated in 24-well plate and allowed to grow for 24 hrs. A viral suspension (~0.5 ml) containing lentivirus (10 μl), growth media (500 μl) and Polybrene (0.5 μl-5 μg/ml) was added to the cells and cultured overnight. Regular growth media was added next day and after 72 hrs GFP expression was visualized. Following this, Puromycin selection was performed (5-10 μg/ml). To obtain higher NMEs expression, GFP positive cells were FACS sorted and were maintained in Puromycin containing growth medium.

### Mutagenesis

NME1 ORF purified plasmid was purchased from GeneCopoeia (EX-M0930-Lv203) along with its vector control (EX-NEG-Lv203). Mutagenesis of NME1 was performed using QuikChange II Site-Directed Mutagenesis Kit (Cat# 200523, Agilent technologies, Santa Clara, USA) for generating P96S (F-5’-CGGGGAGACCAACTCTGCAGACTCCAAGC-3’) and H118F (F-5’-ACAGAATCACTGCCAAATATAATGTTCCTGCCAACTTGTATGCAGA-3’) mutants. PCR conditions used for mutagenesis was 95°C-1 min; 18 cycles of 95°C-50 sec, 60°C-50 sec, 68°C for 6.5 min; 68°C for 7 min. PCR product was transfected and bacterial colonies were screened for positive clones and finally sequence verified.

### NDPK assay

NDPK assay was performed as described previously [17, 64, 65]. Briefly, purified enzyme (EC 2.7.4.6, N2635-100UN, Sigma Aldrich) or lysate (preferably 10 μl) was added to a (preferably 990 μl) reaction mixture containing 83 mM triethanolamine, 0.70 mM thymidine 5’-diphosphate, 2.2 mM adenosine 5’-triphosphate, 0.2 mM β-NADH, 1.1 mM phospho(enol)pyruvate, 16.7 mM magnesium chloride, 66.7 mM potassium chloride, 10 units lactic dehydrogenase and 7 units pyruvate kinase. The reaction was mixed immediately and decrease in absorbance of NADH at 340 nm was then recorded for 5 min. Following this, ΔA_340nm_/minute was calculated using the maximum linear rate for both the Test and Blank group. Units/ml/mg protein was calculated by the following formula:

= [(DA340nm/min Test - DA340nm/min Blank) (Volume of assay) (dilution factor)] / [(6.22) (volume of sample (ml) x concentration mg/ml)]

### 1 and 3-phosphohistidine immunoblotting

Detection of phosphohistidine using 1-phosphohistidine and 3-phosphohistidine antibodies were performed per the previously reported protocol [37]. Briefly, a modified western was performed to preserve phosphohistidine by using basic pH buffers (8-9) and by avoiding heating in sample preparation. For whole cell lysates, 70-100% confluent MDA-MB-231T and MDA-MB-435 cells in 10 cm^2^ dishes were rinsed twice with 5 ml cold TD buffer (TBS −/−, pH 8). Cells were harvested by scraping them in 2X sample buffer, pH 8.8 (5X = 10% SDS, 250 mM Tris-HCl, pH 8.8, 0.02% bromophenol blue, 50% glycerol, 50 mM EDTA, 500 mM DTT). Harvested cells were incubated on ice, sonicated (3-5 × 5 sec bursts) and were clarified by centrifugation (14,000 x *g* for 5-15 min at 4°C). Supernatants were collected and immediately analyzed on freshly prepared Bis-Tris polyacrylamide gels with a modified, pH 8.8 stacking gel (5ml: water-2.975ml, 0.5M Tris-HCl pH8.8-1.250ml, 10% SDS-50μl, Acrylamide-670μl, 10% APS-50μl, TEMED- 5μl) and 12% resolving gels (10ml: water-3.2ml, Acrylamide-4ml, 1.5M Tris-HCl pH8.8-2.6ml, 10% SDS-100μl, 10% APS-100μl, TEMED- 10μl). Samples were resolved at 90-100V for 2-3 hr (4°C) in 1X running buffer (1L, pH 8.5; SDS-1g, Trizma Base-3g, glycine-14.4g, water to 1L) and Proteins were transferred to Immobilon-FL PVDF membranes at 30V for 12-18 hr at 4°C in Transfer Buffer: (1X 4L, pH 8.5, 56.7 g glycine, 4 g SDS, 12 g Trizma Base, 800 ml MeOH, water to 4L). Membrane was incubated for 1 hr at RT in Blocking Buffer (Odyssey Blocking Buffer, TBS -LICOR 927-50000). Membrane was incubated with Primary antibody (Anti-N1-Phosphohistidine-1:1000, MABS1330 or Anti-N3-Phosphohistidine 1:1000, MABS1351, Millipore) diluted in blocking buffer with 0.1% Tween-20, for 1 hr at RT. Membrane was washed thrice for 10 min each with 0.1% TBST. Following this, membrane was incubated with secondary antibody (DyLight-680 goat anti-rabbit IgG, diluted 1: 10,000 in blocking buffer supplemented with 0.1% Tween-20 and 0.01% SDS) for 1hr at RT. Membrane was finally washed with 0.1% TBST thrice and visualized in Multi-application gel imaging system PXi (Syngene). For multiplexing, following 1-pHis detection, blot was incubated in β-actin antibody (1: 10,000, A5441-0.2ML, Sigma Aldrich) overnight and after washing thrice for 10 min each with 0.1% TBST, secondary antibody was added (Alexa Fluor^®^ 568 donkey anti-mouse IgG, diluted 1: 10,000 in blocking buffer supplemented with 0.1% Tween-20 and 0.01% SDS). For total NME, the same blot was stripped (Restore Western Blot-Stripping Buffer, #21059, Thermo Fisher Scientific Inc.) and after blocking, blot was incubated with Primary antibody (NME, sc-343, 1:1000, Santa Cruz Biotechnology) for 1 hr at room temperature. Membrane was washed thrice for 10 min each with 0.1% TBST and incubated with secondary antibody (DyLight-680 goat anti-rabbit IgG, as before) for 1hr at RT. After washing, blot was visualized in Multi-application gel imaging system PXi (Syngene).

### Lysates and protein quantitation

Both MDA-MB-231T and MDA-MB-435 cells were twice washed in 1X Phosphate-buffered saline. Cells were (10 cm^2^ dish) lysed in 100 μl RIPA buffer (20mM Tris-HCl, pH 8.0; 100mM NaCl; 10% Glycerol; 1% NP-40; 0.5% Sodium deoxycholate; 0.1% SDS; Protease Inhibitor Cocktail) added directly on cells and were incubated on ice for 10 min. Cells were scraped and clarified by spinning at 10,000 rpm for 10 min at 4°C. supernatants were collected and protein estimation was performed by BCA analysis (Pierce^®^ BCA Protein Assay Reagent A and B- Prod# 23228). Lysates were frozen at −80°C until use. Equal amount of protein was loaded on Any KD^TM^ Mini-PROTEAN TGX^TM^ Gels (BIO-RAD, Cat# 456-9033).

### Protein expression and purification

Cloning, expression and purification of NME1 and mutant NME1 (P96S and H118F) were performed at Protein Production Core, Frederick National Laboratory for Cancer Research (FNL). All the constructs were His tagged (His6-Hs.NME1, His6-Hs.NME1^P96S^, His6-Hs.NME1^H118F^ for protein purification. To test the quality and amount of protein induction, a microscale induction was performed in *E. coli* cells using 0.5 mM IPTG in rich media (Dynamite) or by auto-induction. All the three proteins produced more purifiable protein using Dynamite expression media. Finally, 100 ml of *E. coli* cell cultures were induced with 0.5 mM IPTG in rich media (Dynamite) and purified by affinity purification using immobilized metal affinity chromatography (IMAC). Affinity purification of proteins using IMAC is based on the interactions between a transition metal ion (Co2+, Ni2+, Cu2+, Zn2+) immobilized on a matrix and specific amino acid side chains such as histidine which exhibits the strongest interaction [66]. All the purified samples were dialyzed into 50 mM Tris, pH 8.0, 150 mM NaCl, 1 mM TCEP. To test the purity of proteins, a coomassie stained 10-20% gradient SDS-PAGE gel of the partially purified proteins was run. Following this all samples were aliquoted and frozen at −80°C.

### 1-phosphohistidine immunofluorescence

Immunofluorescence for the detection of 1-phosphohistidine was performed as described previously [37]. Briefly, MDA-MB-231T and MDA-MB-435 cells were plated (10^4^ cells/well) on chamber slides and allowed to grow for 24 hrs. Cells were washed with PBS (pH 7.4) and fixed in 4% PFA for 20 min. Cells were then washed twice with PBS (pH 7.4) and permeabilized in PBS (pH 9.0) containing 0.1% Triton X-100 at RT for 15 min. Cells were washed thrice in PBS (pH 9.0) and blocked in 4% BSA containing 0.1% TBST, at RT for 30 min. Cells were incubated with primary antibody (Anti-N1-Phosphohistidine antibody SC1-1, #MABS1330, dilution 1:100, Millipore) at RT for 90 min and washed thrice in 0.1% TBST for 5 min. Cells were incubated in secondary antibody (Alexa Fluor-Sheep-anti-rabbit 568 nm, dilution 1:2,000, in TBST containing 1% BSA) at RT for 60 min in the dark. Cells were washed thrice with TBST at RT for 5 min. To stain nuclei, chamber slides were incubated with PBS containing DAPI (1:5,00) for 10 min and washed thrice with PBS. Chamber slide was mounted with a coverslip using mounting media and visualized in confocal microscope under 60X magnification. For the detection of total NME1/2, after primary (1-pHis) incubation was over, cells were washed twice with PBS and were incubated with NME1/2 antibody (sc-465, Santa Cruz Biotechnology, 1:100) overnight in 1% BSA in 0.1% TBST. The secondary antibodies for both NME1/2 (Alexa Fluor-Goat anti-mouse 568 nm) and 1-pHis (Alexa Fluor-Sheep-anti-rabbit 633 nm) were added together.

### Cellular viability

Equal numbers (10,000 cells/well) of MDA-MB-231 (Vector, NME1, NME-2) and MDA-MB-435 (C-100 and H1-177) cells were plated in 96-well plates. Cellular viability after 24 and 48 hrs was measured using AlamarBlue dye (Thermo scientific, USA) by incubating cells with AlamarBlue dye in the growth medium (1:10) for 3 hrs. Viable cells change the color of this dye from blue (non-fluorescent) to red (fluorescent) which was quantitated at 560/590 nm (excitation/emission). Percent viability was calculated using the following formula: % Viability = (Fluorescence of overexpressing cells/Fluorescence of the vector) x 100

### Boyden chamber motility assay

Motility assays were performed as described previously [67]. Briefly, cells were trypsinized and counted using MOXI Z cell counter. Equal number of cells were plated into each upper wells of Boyden chamber (0.1 million/ml; 56 μl) in DMEM. Upper and lower chamber are separated by a coated membrane (Polyvinyl membrane 8 μm, coated overnight with 5 μg/ml of collagen type I and washed in PBS for 30’). These cells were allowed to migrate towards lower wells of the Boyden chamber containing 1% FBS (30 μl, chemoattractant) in a humidified chamber at 37°C and 5% CO_2_ for 4 hrs. Non-migrated cells were wiped off and cells that had migrated to the undersurface of the membrane were fixed and stained with Diff-Quik solutions (Dade-Behring). All the stained and migrated cells were examined microscopically. Representative areas of each well were counted to determine the number of cells that had migrated. Mean (+/− SEM) number of cells migrated (*n* = 4) are plotted in the graph.

### Migration assay

For 2D migration assay 2×10^5^ cells (MDA-MB-231T and MDA-MB-435) were plated per well to 6-well plates in normal growth medium. Cells were grown for 24 hours to attain ~90% confluency. A horizontal line was drawn on the outside of 6-well plate to mark the scratch area. A vertical scratch was made in the center of each well using P200 pipette tip which bisects outer horizontal line. Cells were washed to remove debris and growth medium (0.2% FBS) was added to it. Specific scratch area was photographed using a phase contrast microscope (10X) and marked as time zero (0 hrs). The same area was photographed after 24 hrs and percent area open of each well was calculated using ImageJ. Percent (%) Area Closed was calculated by subtracting area closed at 24 hrs to area closed at 0 hrs for each well.

### *In vitro* histidine protein kinase assay

Recombinant NME1 (WT) and mutant NME1 (P96S and H118F) proteins were purified as per protocol described above. Recombinant SUCLG1 and SUCLG2 proteins (Abnova, H00008801/02; GST tagged) were purchased. For phosphohistidine transfer reaction, wild type NME1 or mutant NME1 (400 ng) was added with either SUCLG1 or SUCLG2 (600 ng) to TMD buffer (20mM Tris-HCl, pH 8.8; 5mM MgCl2; 1mM DTT) containing ATP (0.25 μM). This reaction was incubated for 30’ at room temperature. Reaction was stopped by adding 5x lysis buffer (final 2x) and 1-phosphohistidine western protocol was used for the detection of N1 or N3-phosphohistidine. The same blot was stripped (Restore Western Blot-Stripping Buffer, #21059, Thermo Fisher Scientific Inc.) and processed for detection of SUCLG1/2 using anti-GST antibody (1:2000, ab19256). For detection of phosphoserine, phosphothreonine and phosphotyrosine, the reaction was stopped using 2x loading sample buffer and samples were heated at 95°C for 5 min. Samples were resolved on Any KD^TM^ Mini-PROTEAN TGXTM Gels and, after transfer, membranes were incubated with p-Serine (AB1606, 1:1000, Millipore), P-Threonine (AB1607, 1:1000, Millipore) or p-Tyrosine (05-321, 1:1000, Millipore) antibody. Following stripping, anti-GST and NME proteins were detected. MDA-MB-231T cell lysate was used as positive control.

### Blue native polyacrylamide gel electrophoresis (BN PAGE)

NativePAGE™ Gels (Pre-cast NativePAGE™ Novex 4-16% (v/v) Bis-Tris, BN1002BOX) were purchased from Thermo Fisher Scientific Inc. It is based on the Blue Native Polyacrylamide Gel Electrophoresis (BN PAGE) technique, where in Coomassie G-250 was used to confers a net negative charge on proteins while maintaining the proteins in their native state (without any protein denaturation). Recombinant protein samples (WT-NME1, NME^P96S^ and NME1^H118F^-8μg) were first mixed with or without ATP (1μM) and incubated for 30’ at room temperature. The reaction was stopped using sample buffer (final-1x) and samples were loaded on the gel. Gel was run using Dark Blue Cathode (1X) and Anode Buffer (1X) with near neutral pH (7.5) at 150 V for 1.5 hrs and 250 V for 30’ at 4°C. NativeMark™ Unstained Protein Standard was used as protein marker. Following this, gels were destained in Methanol 40%, Acetic Acid 10% overnight. Images were taken in ChemiDoc Touch imaging system (Bio-Rad).

### Statistical analyses

All experiments were repeated at least three times unless noted. For motility, migration and NDPK assays, statistical significance was calculated by a Student's t test or 1-way ANOVA.

## SUPPLEMENTARY MATERIALS FIGURES



## Abbreviations

NDPK: Nucleoside Diphosphate Kinase
HPK: Histidine protein kinase
AWD: Abnormal Wing Discs
KSR: Kinase Suppressor of Ras
