# Supplementary material for: The relationship of NM23 (NME) metastasis suppressor histidine phosphorylation to its nucleoside diphosphate kinase, histidine protein kinase and motility suppression activities
Source: Oncotarget. 2017 Dec 31;9(12):10185–202. doi: 10.18632/oncotarget.23796 (PMC5828198; doi:10.18632/oncotarget.23796)
Supplement: Supplementary file 1 [file oncotarget-09-10185-s001.pdf]

The relationship of NM23 (NME) metastasis suppressor histidine phosphorylation to its nucleoside diphosphate kinase, histidine protein kinase and motility suppression activities

SUPPLEMENTARY MATERIALS

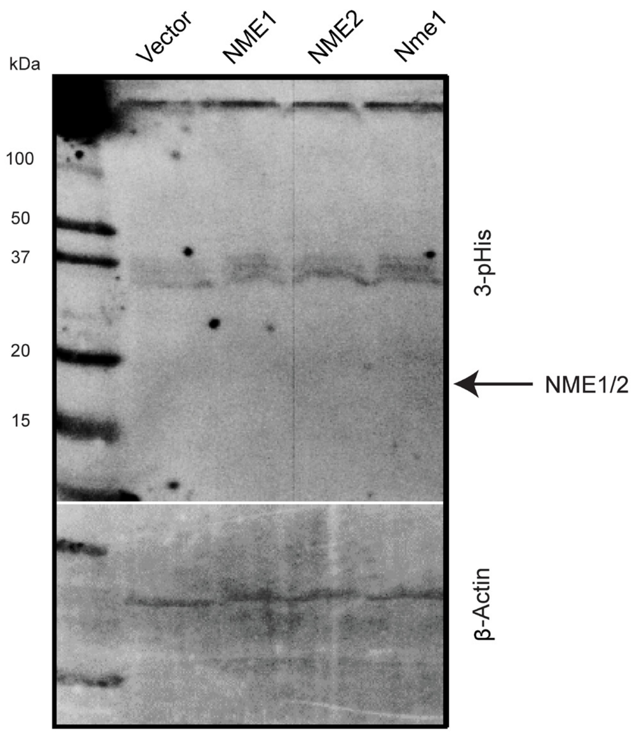

Supplementary Figure 1: NME1/2 is not phosphorylated at N3-histidine. Cell lysates prepared from MDA-MB-231T cells transfected with vector construct, Flag-tagged human NME1, NME2 or mouse ortholog Nme1 were assessed for 3-phosphohistidine (3-pHis) levels using anti-N3-phosphohistidine antibody. β-actin was used as loading control.

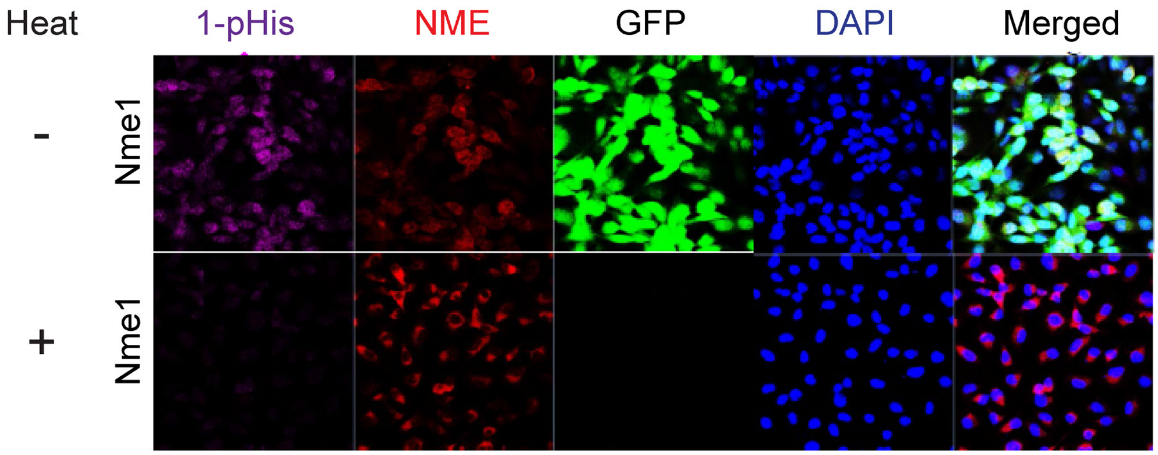

Supplementary Figure 2: 1-Phosphohistidine immunofluorescence staining is heat sensitive. Nme1 overexpressing (GFP co-expressed) MDA-MB-231T cells were plated in chamber slides and cultured for 24 hrs. To assess the specificity of 1-pHis staining (negative controls), following PFA fixation, chamber slides were boiled in 0.01 M citrate buffer for 10 min. Following this, 1-pHis and NME immunostaining was performed as per the protocol. Loss of 1-pHis staining was apparent, highlighting its heat sensitive nature, while Nme1 expression was not heat sensitive.

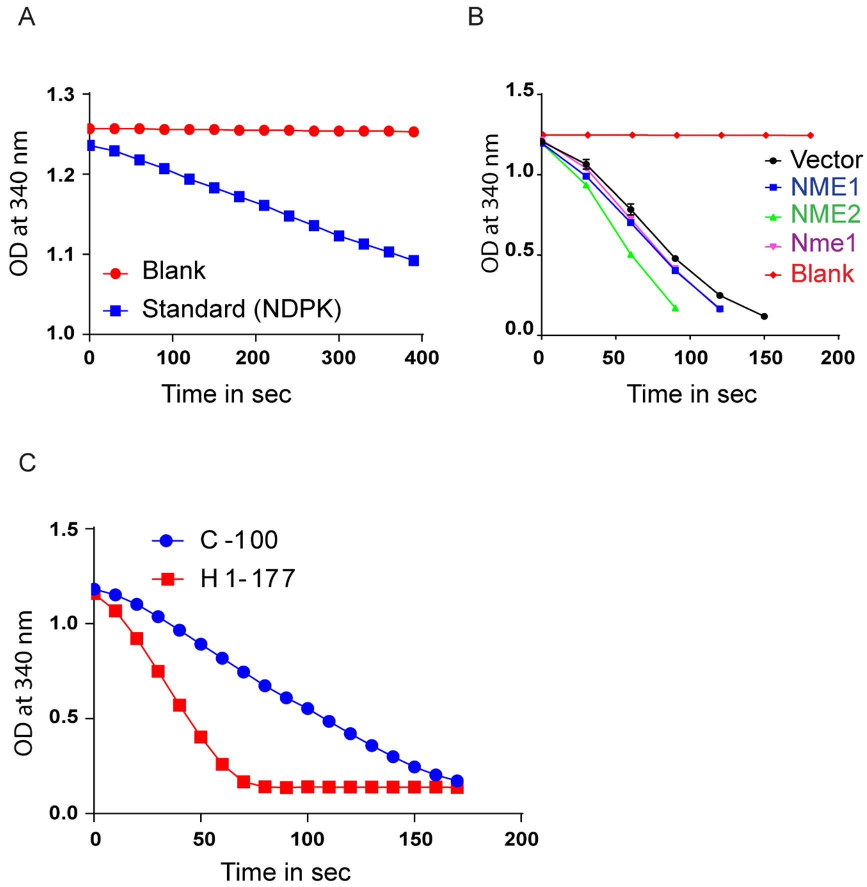

Supplementary Figure 3: NDPK activity time course. The NDPK activity of cell lysates was quantified by a spectrophotometric assay for the formation of NAD (Oxidized) from NADH (reduced) leading to the loss of OD at 340 nm. (**A**) Time course showing Loss of OD at 340 after addition of commercially available pure NDPK (EC 2.7.4.6). (**B**) Time course showing loss of OD at 340 after addition of equal volume (5 µl) of MDA-MB-231T cell lysates of Vector, and NME1, 2 and Nme1 overexpression. (**C**) Time course in MDA-MB-435 cell lysates of Vector (C100), and NME1 overexpression (H1-177).

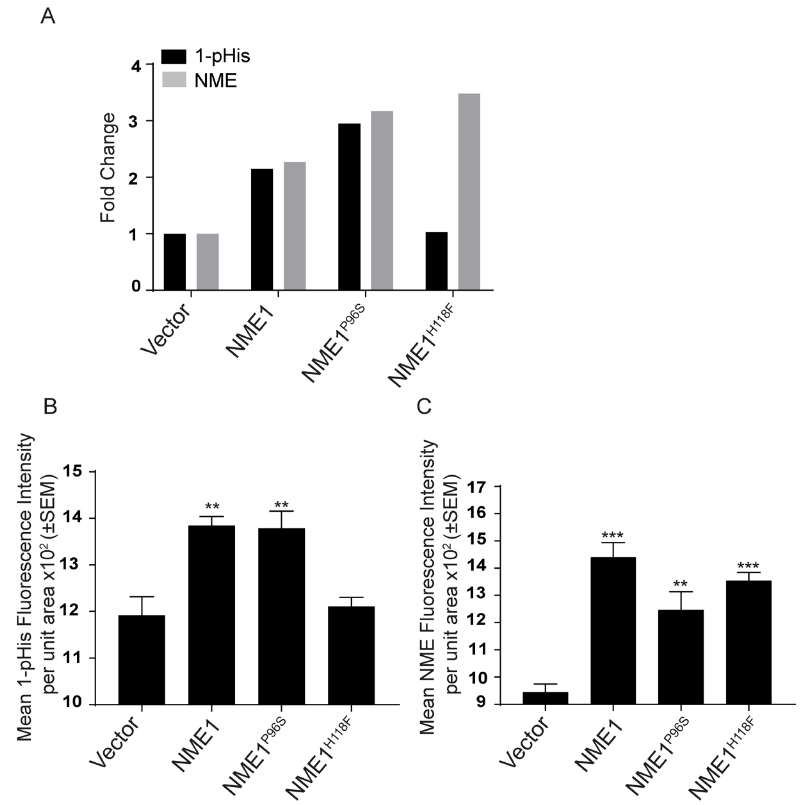

Supplementary Figure 4: NME1^{H118F} is deficient in 1-pHis (Figure 7A and B quantitation). MDA-MB-231T cells stably expressing a vector, wild type NME1 or its P96S or H118F (NME1^{P96S} and NME1^{H118F}) mutations were quantitated for 1-pHis levels by western blotting and immunofluorescence (Figure 7A and B). (**A**) Expression of 1-pHis and NME (top band) in western blot (Figure 7A) was quantitated using imageJ software and presented as fold change over vector control. Quantitation of 1-pHis (**B**) and NME (**C**) expression by immunofluorescence (Figure 7B) in the above cells is plotted as Mean Fluorescence Intensity per unit area (±SEM). 1-way ANOVA was used for assessing significance.

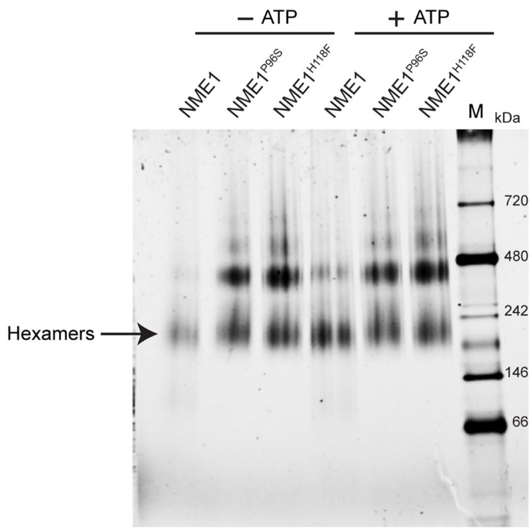

Supplementary Figure 5: Wild type NME1 and side directed mutants (NME1^{P96S} and NME1^{H118F}) form hexamers. Partially purified protein samples (WT-NME1, NME^{P96S} and NME1^{H118F}-8µg) were first mixed with or without ATP (1µM) and were incubated for 30' at room temperature. Samples were loaded on Pre-cast NativePAGE™ Novex 4–16% (v/v) Bis-Tris Gels and were run at 150 V for 1.5 hrs and 250 V for 30' at 4 °C. Gels were destained overnight and photographed. Both Wild type (NME1) and mutants (NME^{P96S} and NME1^{H118F}) formed hexamers (highlighted by the arrow).

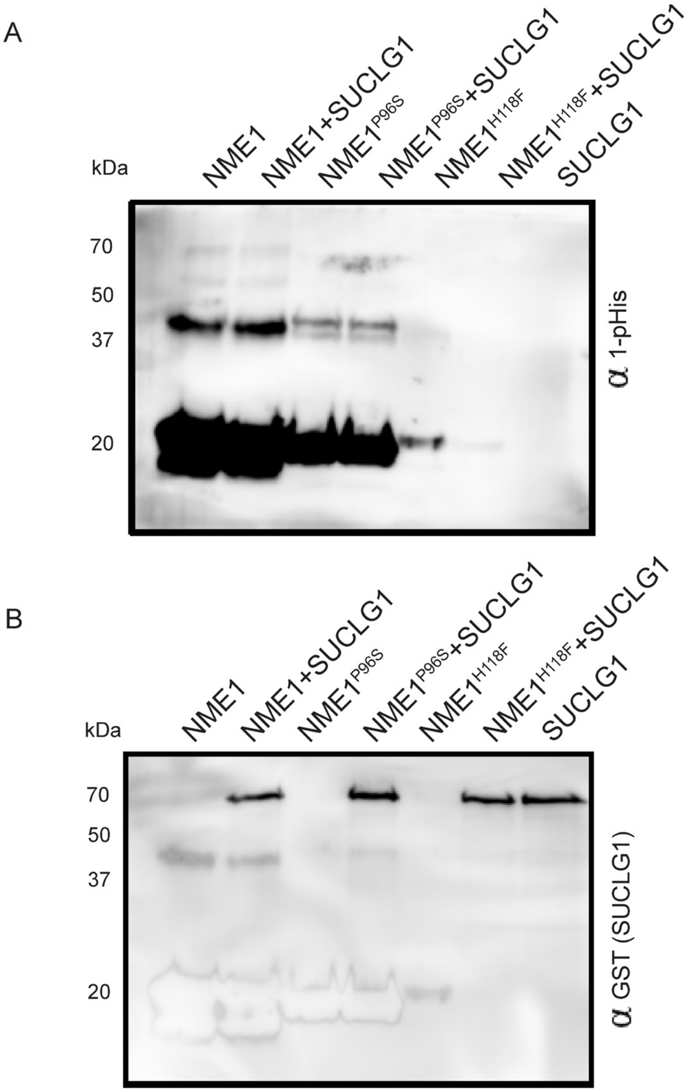

Supplementary Figure 6: SUCLG1 is not a substrate of NME1 for phosphohistidine transfer. To assess HPK activity using SUCLG1 as substrate, the partially purified proteins of NME1, $NME1^{P96S}$ or $NME1^{H118F}$ (400 ng) were incubated with SUCLG1 (600 ng) in TMD buffer containing ATP for 30' at room temperature. Reaction was stopped by adding 5x lysis buffer and 1-phosphohistidine detection was performed (**A**). (1-pHis band at ~20 kDa and ~37 kDa are histidine phosphorylated monomer and dimer of NME1). Following stripping, SUCLG1 (GST tagged) was detected using anti-GST antibody (**B**).

A

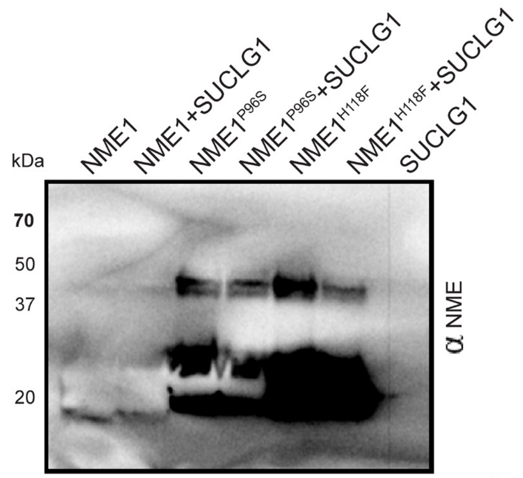

B

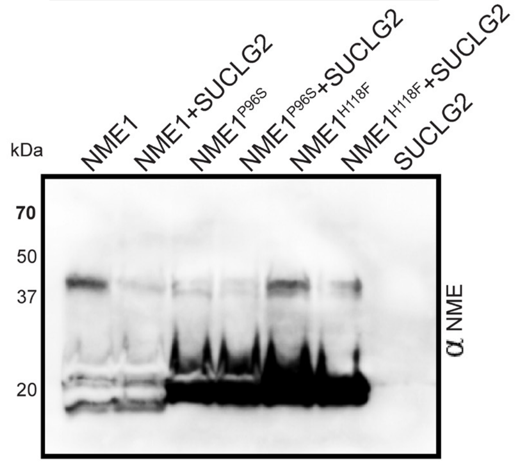

Supplementary Figure 7: Reprobing of SUCLG1 and SUCLG2 blot with NME antibody. To assess if autophosphorylated NME1 (1-pHis) forms any band at 70 kDa by oligomerization, 1-pHis blot of both SUCLG1 (**A**) and SUCLG2 (**B**) were stripped and reprobed with NME antibody. It can be clearly seen from the blot that NME forms predominantly monomer (~20kDa) and dimer (~40kDa). There is no detectable band at 70kDa (highlighted by bold).

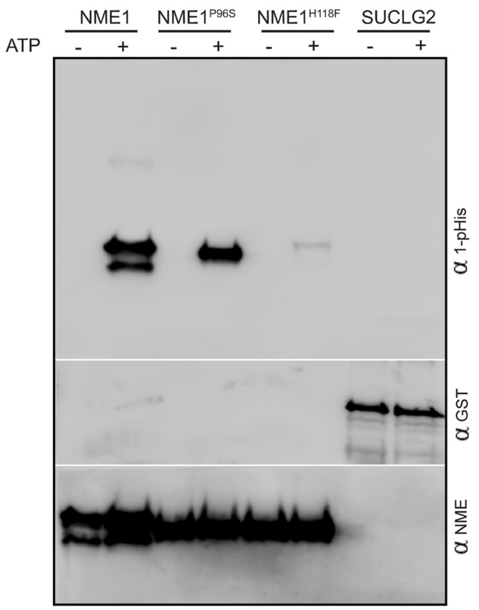

Supplementary Figure 8: NME1 autophosphorylation at 1-pHis is ATP dependent. NME autophosphorylation at Histidine 118 position (1-pHis) was assessed with and without ATP. Partially purified proteins of NME1, NME1[P96S], NME1[H118F] (400 ng) and SUCLG2 (600 ng) were incubated with and without ATP in TMD buffer for 30' at room temperature. Reaction was stopped by adding 5x lysis buffer and 1-phosphohistidine western protocol was used for the detection of 1-pHis. After stripping, SUCLG2 (GST tagged) and NME were detected by anti-GST and NME antibodies, respectively.

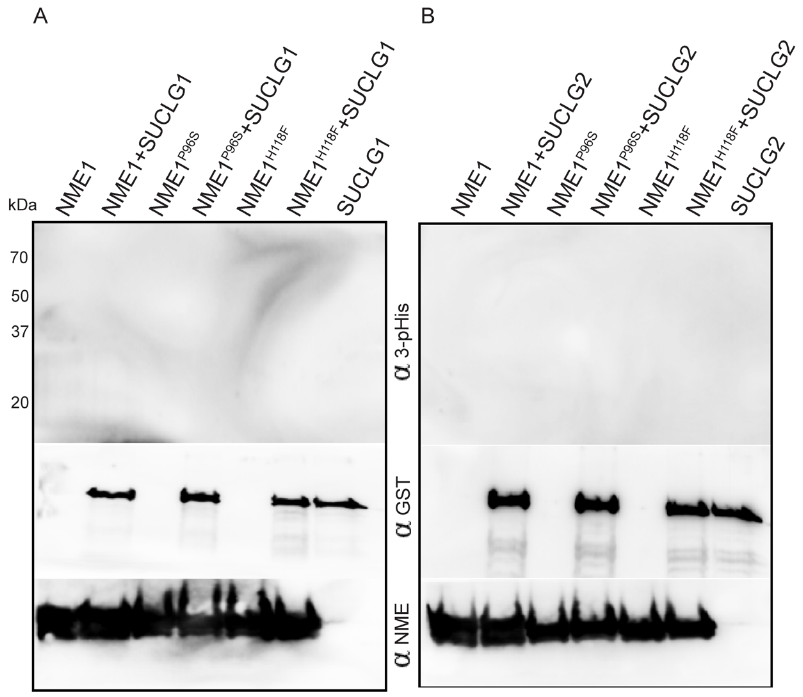

Supplementary Figure 9: No phosphotransfer at N3-histidine of SUCLG1 and SUCLG2 by NME1. To assess HPK activity on N3-histidine of SUCLG1 and SUCLG2 by NME1, the partially purified proteins of NME1, NME1[P96S] or NME1[H118F] (400 ng) were incubated with (**A**) SUCLG1 or (**B**) SUCLG2 (600 ng) in TMD buffer containing ATP for 30' at room temperature. Reaction was stopped by adding 5x lysis buffer and phosphohistidine western protocol was used for the detection of 3-pHis. Following stripping, SUCLG1/G2 (GST tagged) was detected by anti-GST antibody and total NME was detected using NME antibody.

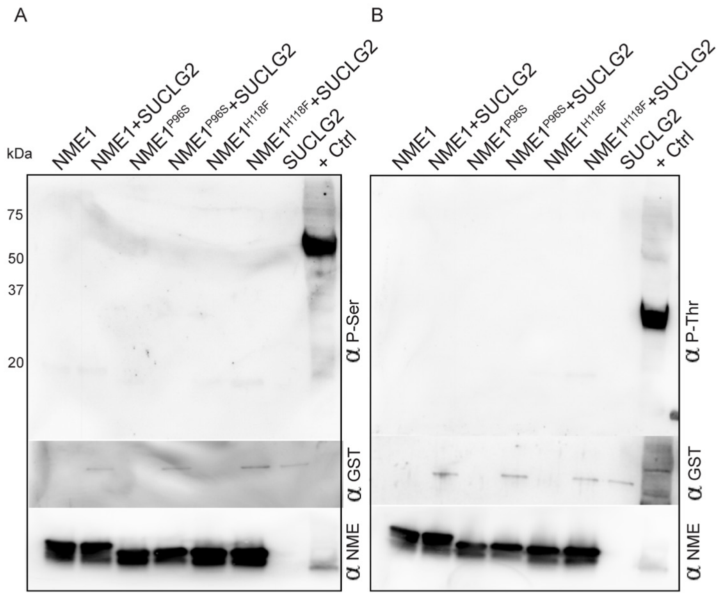

Supplementary Figure 10: No phosphotransfer between NME1 and SUCLG2 at serine and threonine residues. To assess NME-HPK activity on Serine or Threonine of SUCLG2, the partially purified proteins of NME1, $NME1^{P96S}$ or $NME1^{H118F}$ (400 ng) were incubated with SUCLG2 (600 ng) in TMD buffer containing ATP for 30' at room temperature. Reaction was stopped by adding 2x loading Sample buffer and samples were heated at 95°C for 5 min. Samples were resolved on Any KD™ Mini-PROTEAN TGX™ Gels and after transfer, membranes were incubated with phosphoserine (**A**) or phosphothreonine (B) antibody. Following stripping, anti-GST and NME proteins were detected. (+ Ctrl - MDA-MB-231T cell lysate was used as positive control).

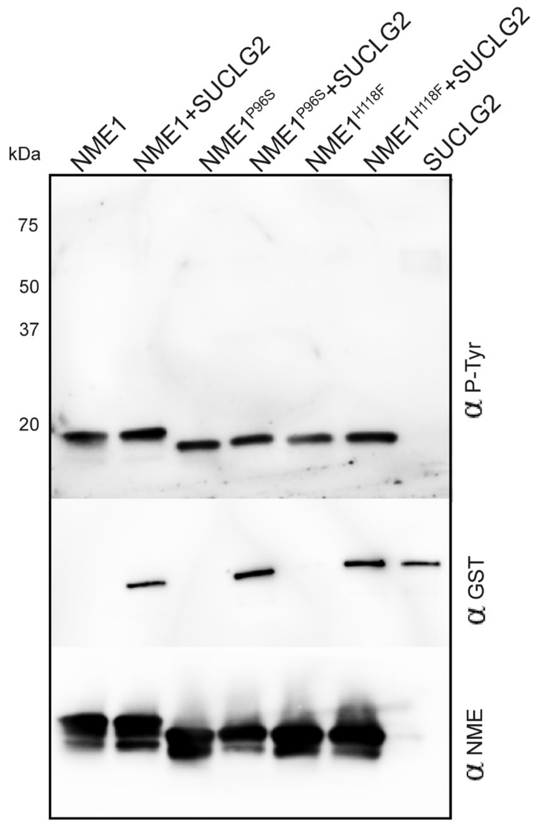

Supplementary Figure 11: No phosphotransfer between NME1 and SUCLG2 at tyrosine residues. To assess NME-HPK activity on Tyrosine of SUCLG2, the partially purified proteins of NME1, NME1^{P96S} or NME1^{H118F} (400 ng) were incubated with SUCLG2 (600 ng) in TMD buffer containing ATP for 30' at room temperature. Reaction was stopped by adding 2x loading Sample buffer and samples were heated at 95°C for 5 min. Samples were resolved on Any KD™ Mini-PROTEAN TGX™ Gels and after transfer, membranes were incubated with phosphotyrosine antibody. Following stripping, anti-GST and NME proteins were detected.
